# Exploring learner acceptance and experience in MOOCs among higher vocational students from a TAM perspective: a case study of college English course

**DOI:** 10.3389/fpsyg.2025.1643166

**Published:** 2025-11-28

**Authors:** Shuhua Hou

**Affiliations:** School of Higher Vocational Education, The Open University of Sichuan, Chengdu, China

**Keywords:** technology acceptance model (TAM), MOOCs, learning experience, higher vocational education, structural equation modeling (SEM)

## Abstract

This study investigates higher vocational students’ acceptance of a College English MOOC through the technology acceptance model (TAM). A survey of 434 students was analyzed using structural equation modeling (SEM). The findings confirm core TAM relationships: perceived ease of use (PEOU) significantly enhances perceived usefulness (PU), which in turn fosters a positive attitude toward use (ATU) and increases intention to use (IU). Furthermore, IU positively impacts the learning experience (LE). A key discovery is the negative effect of ATU on LE, suggesting that initial attitude alone does not guarantee a positive experience, which may be more critically shaped by course content and instructional design. This study enriches the TAM literature within vocational education and provides practical insights for developing MOOCs that better meet the needs of vocational learners.

## 1 Introduction

### 1.1 Background and significance

The rapid advancement of educational technology has revolutionized language learning, with Massive Open Online Courses (MOOCs) emerging as a transformative force in global education by democratizing access to multilingual resources and interactive platforms (Altalhi, 2021). However, the potential of MOOCs is often hampered by low completion rates, underscoring the critical need to understand the psychological mechanisms that drive sustained learning engagement in online environments (Kuo et al., 2021). As an English teacher at a higher vocational institution, I have recognized the necessity of adapting to these changes to enhance teaching effectiveness. While existing research has examined MOOC integration in traditional higher education through blended learning frameworks (de Lima Guedes et al., 2022), vocational education contexts remain underexplored despite their unique pedagogical needs (Margaryan et al., 2015). This gap is particularly salient in vocational education, where teachers’ professional development often prioritizes practical teaching skills over research engagement (Hagedoorn et al., 2025). Furthermore, vocational learners typically require heightened self-regulation to succeed in the less-structured MOOC environment (Alonso-Mencía et al., 2020; Wong et al., 2019).

It is within this context of vocational education that our study is situated. Specifically, technical and vocational education and training (TVET), with its employment-oriented nature, increasingly relies on digital technologies like MOOCs for high-quality development (Ye et al., 2024). Yet, research on MOOC acceptance in TVET has predominantly focused on professional courses, leaving a significant gap in understanding its application to public fundamental courses like College English—a subject critical for students’ career readiness.

To address this gap, we developed a provincial-level quality MOOC on College English hosted on Xueyin Online (China’s major MOOC platform). Over nine sessions, the course has attracted 13,587 enrollments, with 4,165 current learners (primarily freshmen aged 19–20), demonstrating its practical significance for vocational education.

### 1.2 Research gaps and theoretical positioning

The technology acceptance model (TAM) (Davis, 1989) provides a robust framework for understanding MOOC adoption. While classical TAM establishes perceived ease of use (PEOU) and perceived usefulness (PU) as key determinants of technology acceptance (Davis, 1989; Venkatesh et al., 2003), three critical limitations persist in vocational MOOC contexts.

First, while studies like that of Kuo et al. (2021) have explored mediating factors like online academic hardiness between self-efficacy and engagement, the specific mediation pathway from PEOU to attitude via PU within the TAM framework remains underexplored in vocational settings. Second, structural institutional factors (e.g., state-employer relations) are critically understudied in vocational education and training systems, as demonstrated by Egypt’s path-dependent institutional constraints (Soliman, 2025). Third, the role of self-regulated learning (SRL) is crucial in MOOCs (Alonso-Mencía et al., 2020; Wong et al., 2019), yet how credential-oriented motivation interacts with the core TAM constructs to influence the learning experience for vocational students is unclear.

While TAM posits universal PU/PEOU effects (Venkatesh et al., 2003), vocational learners’ credential-seeking motives may weaken PU’s influence—a phenomenon observed in traditional e-learning but untested in MOOCs where micro-credentials intensify credential salience. Recent studies confirm employers perceive micro-credentials as “flexible but outcome-driven” (Bruguera et al., 2025), suggesting learners may prioritize badge acquisition over holistic skill benefits, thereby suppressing PU’s traditional performance-enhancement logic.

**Figure 1:**
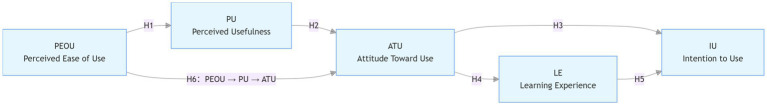
Conceptual framework.

To explain higher vocational students’ continuous use intention of College English MOOCs, this study is guided by the TAM-ECT integrated framework (Ye et al., 2023). As depicted in Figure 1, our model extends classical TAM by (a) inserting PU as a mediator between PEOU and attitude toward use (ATU), testing a key psychological mechanism akin to the mediation effects explored by Kuo et al. (2021), and (b) positioning learning experience (LE) as an outcome (not antecedent) of ATU, reflecting vocational learners’ attitude-driven experience formation, which is closely tied to their capacity for self-regulation in MOOCs (Wong et al., 2019).

While Davis (1989) postulated direct PEOU → ATU effects, recent vocational studies (e.g., Soliman, 2025) found institutional mandates may override usability perceptions. Our PU mediation hypothesis (H6) thus extends Wheelahan and Moodie’s (2025) credentialism theory by testing whether vocational learners prioritize utility over ease, while also accounting for the need to foster engagement and self-regulation as highlighted in the broader MOOC literature (Alonso-Mencía et al., 2020; Kuo et al., 2021).

### 1.3 Hypotheses development

Following Wheelahan and Moodie (2025), “credential-oriented motivation” here refers to vocational learners’ prioritization of certification over intrinsic learning experience, measured by enrollment records correlating with diploma requirements. Based on the conceptual framework (Figure 1), we propose:

*H*1: Perceived ease of use (PEOU) positively influences perceived usefulness (PU). Students who find the MOOC platform easy to use are more likely to perceive the MOOC as beneficial for their learning.

*H*2: Perceived usefulness (PU) positively influences attitude toward use (ATU), particularly when content aligns with vocational career goals. Students who perceive the MOOC as beneficial for their learning are more likely to have a favorable attitude toward the use of the MOOC platform.

*H*3: Attitude toward use (ATU) positively influences Intention to Use (IU). Students who have a favorable attitude toward the use of the MOOC platform for the course are more likely to have the intention to use the MOOC platform in the future.

*H*4: Attitude toward use (ATU) has a significant influence on Learning Experience (LE). Students with a positive attitude toward the use of the MOOC platform are more likely to have a positive learning experience, which is foundational for fostering the self-regulated learning behaviors essential in MOOCs (Wong et al., 2019).

*H*5: Learning experience (LE) positively influences Intention to Use (IU). Students who have a positive learning experience with the MOOC platform are more likely to have the intention to use the MOOC platform again in the future.

*H*6: PEOU exerts an indirect effect on ATU through PU (examined via SEM’s indirect effect estimation). This mediation hypothesis aims to clarify a key psychological pathway in the acceptance of MOOCs by vocational learners, contributing to a more nuanced understanding of online learning engagement (Kuo et al., 2021).

### 1.4 Key contributions

To bridge this gap and provide actionable insights for vocational education, this study will make the following key contributions:

1. Theoretical advancements: This study establishes the indirect influence of perceived ease of use (PEOU) on attitude toward use (ATU) via perceived usefulness (PU) in the context of vocational MOOCs and clarifies how usability perceptions translate into adoption attitudes—a mechanism previously underexplored in cross-sectional vocational settings. Notably, it tests a deviation from Davis’s (1989) original technology acceptance model, examining whether PU fully mediates the PEOU-ATU relationship, which may imply that institutional factors in vocational training override direct usability perceptions.
2. Practical implications: We derive evidence-based design principles for vocational MOOC platforms, such as adaptive scaffolding strategies to reduce cognitive load and competency-based assessment frameworks aligned with industry needs. These findings provide educators and platform developers with direct guidance to optimize usability and better connect instructional design to vocational outcomes.
3. Methodological Rigor: We advance vocational education research by employing structural equation modeling (SEM) to systematically test the hypothesized mediation pathway (PEOU → PU → ATU) in a cross-sectional dataset. Confirmatory factor analysis (CFA) validated the measurement model fit and discriminant validity of constructs. Global fit was assessed via established indices (CFI > 0.90, RMSEA < 0.10). This approach ensures transparent, replicable, and theoretically grounded conclusions about the mechanisms linking perceived ease of use (PEOU) to attitude toward use (ATU) and learning outcomes in MOOCs.

## 2 Literature review

### 2.1 TAM in vocational contexts: theoretical tensions

#### 2.1.1 Divergent effect sizes

The Technology Acceptance Model (TAM) (Davis, 1989) establishes perceived usefulness (PU) and perceived ease of use (PEOU) as universal predictors of technology adoption. As one of the most influential theories in information systems research, the parsimonious TAM has been extensively extended, notably by the incorporation of social influence and cognitive processes in TAM2 (Venkatesh and Davis, 2000) and the unified UTAUT framework (Venkatesh et al., 2003). Scherer et al. (2019) confirmed these core relationships through a meta-analytic structural equation model of 114 empirical studies, while revealing significant heterogeneity across educational contexts, underscoring the need for vocational-specific analyses.

While validated in settings from enterprise software (Venkatesh and Davis, 2000) to academic e-learning (Šumak et al., 2011), recent studies highlight critical gaps in vocational and learner-centered contexts. Prior research suggests vocational learners prioritize utility over ease of use. Scherer et al. (2019) reported weaker perceived ease of use (PEOU) effects in applied contexts, while Hoi and Mu (2021) found students valued teachers’ resource guidance more than technical training, further supporting the dominance of pragmatic utility in applied settings. Consistent with these findings, Niu and Wu (2022) examined vocational college students’ online learning during the COVID-19 pandemic and observed that perceived usefulness was a stronger predictor of creativity than ease-of-use perceptions—reinforcing the centrality of utility in vocational learners’ technology acceptance.

Research on technology acceptance has progressively emphasized the importance of cultural context. Srite and Karahanna (2006) were instrumental in this shift, moving beyond demographic moderators to establish espoused national cultural values as key moderators. They demonstrated that uncertainty avoidance significantly strengthens the effect of social norms on behavioral intentions.

This theoretical framework is empirically supported by the cross-cultural study of Tarhini et al. (2015), who compared British and Lebanese university students. Their findings, which revealed significant differences in factors like social norms between the two cultural groups, provide strong evidence that national culture systematically influences the technology acceptance process.

In summary, the work of Srite and Karahanna (2006) and Tarhini et al. (2015) collectively confirm that cultural dimensions, particularly uncertainty avoidance, are critical for a complete understanding of technology adoption across different societies.

#### 2.1.2 From language learning specificity to MOOC adaptation

The transition of language education to the MOOC format reveals a core tension: the interactive, practice-intensive nature of language acquisition conflicts with the standardized, mass-delivery model of early MOOCs. This misalignment underscores a utility-driven pattern where learners seek tangible proficiency gains, a need that generic MOOC structures often fail to meet.

Evidence of this mismatch is clear at both macro and micro levels. Macroscopically, despite significant growth in course numbers, language MOOCs in Mainland China face challenges of “resource wastage” and plummeting enrollments (Yin and Ma, 2025), indicating that scalability alone does not guarantee effectiveness or sustainability. This utility gap is further examined at the micro-level: Jiang and Peng (2023) found that within an English language MOOC, only deep cognitive engagement—fueled by learner autonomy—significantly predicted academic success, not mere behavioral activity. This confirms that passive content consumption is insufficient for language learning.

Consequently, research has shifted from simply documenting language MOOCs to critically evaluating their pedagogical design. As Sallam et al. (2020) highlighted, a key trend is the search for models that move “beyond the xMOOC/cMOOC dichotomy,” signaling a concerted effort to design language MOOCs that better support the autonomous, cognitively engaging practice essential for language proficiency. This evolution marks a critical step toward aligning language MOOC design with the utility-driven demands of learners.

### 2.2 Vocational MOOCs: a structural remedy

The rapid adoption of Massive Open Online Courses (MOOCs) in professional education necessitates examining how the learning experience (LE) operates within this distinct ecosystem. The structural features of MOOCs can precisely address the TAM limitations identified above.

We posit that employer-mediated engagement acts as a key structural compensator for potential deficits in perceived ease of use (PEOU).

#### 2.2.1 Employer-mediated engagement as a PEOU compensator

Research on technology acceptance in organizational contexts consistently demonstrates that external institutional factors can significantly alter the traditional TAM pathways, which primarily focus on individual perceptions. A key theoretical extension is the role of subjective norm from the Theory of Planned Behavior, which has been integrated into models like UTAUT as social influence. Venkatesh and Davis (2000) in their seminal work on TAM2 found that subjective norm (a construct capturing social pressures from peers, superiors, and the organization) can have a direct effect on users’ perceptions of usefulness, especially in mandatory settings.

This logic translates directly to vocational MOOCs. Workplace accountability mechanisms—such as supervisor recognition or linking course completion to career advancement—act as powerful manifestations of subjective norm (Bruguera et al., 2025). These mechanisms may amplify the relationship between the learning experience and a positive attitude towards the MOOC, effectively compensating for potential shortcomings in individual-level perceptions of ease of use. When a learning tool is deemed essential for career progression, the external motivation driven by the employer can override intrinsic usability concerns, a phenomenon supported by research on mandatory system use (Brown et al., 2002).

#### 2.2.2 Credential-driven patterns: the PU catalyst

Vocational learners exhibit distinct behavioral patterns driven by certification goals. The phenomenon of “certification sprints”—intensive engagement aimed specifically at credential acquisition (Ha et al., 2022)—is well-documented in the literature on MOOC learner motivations and behaviors. Seminal research by Pant et al. (2021) in their study of MOOC dropout factors explicitly identified “desire for certification” as a primary motivator for a significant subset of learners who actively complete courses. This goal-directed behavior is particularly salient in vocational contexts. The pursuit of a credential can fundamentally reframe the construct of perceived usefulness (PU). Rather than being solely derived from the intrinsic value of the learning content, PU is significantly augmented by the instrumental value of the certificate itself—its utility for career advancement, salary increase, or professional certification. This aligns with the broader concept of extrinsic motivation in learning theory, where external rewards drive engagement (Deci et al., 1999). In essence, for the vocational learner, the MOOC’s usefulness is not just about learning depth but about the efficiency and credibility of the credentialing process.

#### 2.2.3 Peer validation: bridging the authenticity gap

Unlike academic MOOCs where forum discussions may center on theoretical debate, vocational learners are hypothesized to utilize peer interactions primarily to benchmark their practical competencies. This process is effectively captured by recent research on the social dynamics of peer assessment. Specifically, a study by Zhao et al. (2025) demonstrated that group-oriented metacognitive scaffolding helps online learners co-construct shared evaluation criteria, fostering a collective understanding of competent performance.

This social epistemic process is further reinforced by individual cognitive benefits. The act of providing detailed feedback itself sharpens the assessor’s own critical judgment, a phenomenon well-documented in earlier research (Lundstrom and Baker, 2009).

Consequently, in a vocational language MOOC, this dual-level validation—both social and individual—directly bridges the “authenticity gap.” For instance, when learners evaluate the persuasiveness of a peer’s sales pitch, they are actively negotiating professional standards. This peer-mediated process serves as a powerful proxy for real-world feedback, grounding the learning experience in socially-constructed authenticity (Lave and Wenger, 1991).

## 3 Research methodology

This study employed a quantitative design to examine the interrelationships among Technology Acceptance Model (TAM) constructs in Chinese vocational college students’ MOOC engagement. The design aligns with recommendations for TAM-based studies in educational technology (Venkatesh and Davis, 2000). Key constructs were measured using adapted scales, including a “College English Learning Attitude Scale” (Hong et al., 2020), whose reliability (Cronbach’s *α*) and validity (CFA) were verified.

### 3.1 Participants

*Sample:* 434 students (valid responses) were recruited via the *College English MOOC* platform, exceeding the estimated requirement of 400 for SEM analysis (Kline, 2023). Therefore, once I saw that the number of responses had met the requirement, I immediately closed the questionnaire.

A random sampling strategy was employed to ensure the representativeness of the target population (vocational college students enrolled in the College English MOOC). Specifically, an automated randomization function within the WJX platform^1^ was utilized to distribute survey links equally across all active MOOC classes, thereby minimizing selection bias.

*Procedure:* The data collection procedure consisted of the following steps:

- *Recruitment and informed consent*: An invitation containing the study’s purpose, estimated completion time, and a statement ensuring anonymity and voluntary participation was posted on the MOOC platform’s announcement board. Participants were required to actively check a box indicating their digital consent before they could proceed to the questionnaire.
- *Survey administration:* The survey was hosted on the WJX platform (a Chinese platform analogous to Qualtrics). Upon providing consent, participants accessed the anonymous online questionnaire, which took approximately 3–5 min to complete.
- *Data collection period:* The survey window remained open for a period of 5 days to ensure adequate opportunity for participation.
- *Data screening*: Upon closure, the dataset was downloaded and screened. Responses that were incomplete or exhibited straight-line patterns (e.g., identical answers for all items) were excluded, resulting in the final 434 valid responses.

*Ethics*: Approved by Sichuan Huaxin Modern Vocational College (#JG2024004Z), complying with APA ethical guidelines. Participants could withdraw anytime without penalty, and data were stored on password-protected servers compliant with China’s Data Security Law of the People’s Republic of China (2021).

### 3.2 Survey instrument

*Dimensions*: 24 items across 5 TAM constructs:

*Perceived ease of use (PEOU)*: 4 items (e.g., “*The MOOC platform is easy to navigate.”*)
*Perceived usefulness (PU)*: 5 items (e.g., “*MOOCs improve my English proficiency.”*)
*Attitude toward use (ATU)*, *Intention to Use (IU)*, *Learning Experience (LE)*: 5 items each.

*Validation*:

*Content validity*: 3 experts in educational technology and English language teaching were consulted to review the initial draft of the questionnaire. Their feedback was incorporated to refine the questions, ensuring clarity, relevance, and appropriateness.
*Scale anchors*: 5-point Likert (1 = *Strongly Disagree* to 5 = *Strongly Agree*).

### 3.3 Analytical procedures

We adopted a quantitative research design to systematically examine the relationships among TAM constructs and vocational MOOC acceptance. A randomized online survey (N = 434 valid responses) used 5-point Likert scales to measure:

- Core TAM constructs (PEOU, PU, ATU, and IU).
- Contextualized learning experience (LE) metrics.

All TAM items were adopted from Venkatesh and Davis (2000) with established factor structures. CFA was directly applied as our vocational education context aligns with their workplace technology adoption setting. Data were analyzed through a two-step process:

- *Confirmatory factor analysis (CFA)*: Validated the measurement model to ensure the reliability and validity of the TAM and LE scales.
- *Structural equation modeling (SEM)*: Tested hypothesized direct and indirect pathways (e.g., the mediating role of PU in the relationship between PEOU and ATU). SEM was chosen for its ability to simultaneously estimate multiple relationships and quantify indirect effects—aligning with our goal to examine how PEOU influences ATU via PU (H6). H6 was grounded in classic TAM extensions (Venkatesh and Davis, 2000), which first validated PU as a critical mediator between PEOU and ATU, and the unified framework of UTAUT (Venkatesh et al., 2003), which positions PU as a key driver of technology adoption attitudes.

**Statistical tools:** Statistical analyses were performed using SPSSAU (Version 2023). Descriptive statistics and reliability coefficients (e.g., Cronbach’s *α*) were calculated first. Confirmatory factor analysis (CFA) and structural equation modeling (SEM) were then conducted to test the hypothesized relationships.

### 3.4 Scale validation

- *Reliability*: All constructs demonstrated high reliability, exhibiting excellent internal consistency (α = 0.993, Table 1), surpassing the threshold of 0.7 (Nunnally and Bernstein, 1994).

**Table 1:** Reliability coefficients for all constructs.

Number of variables	Sample size	Cronbach α
24	434	0.993

The Kaiser–Meyer–Olkin (KMO) measure and Bartlett’s test of sphericity were conducted to assess data suitability for factor analysis. A KMO value > 0.9 and a significant Bartlett’s test (*p* < 0.05) were required (Kaiser, 1974). The KMO measure (0.979) and Bartlett’s test (*p* < 0.001) confirmed sampling adequacy for factor analysis (see Table 2).

**Table 2:** Sampling adequacy tests.

KMO		0.979
Bartlett’s test of sphericity	Approximate chi-square	20988.421
	*df*	276
	*p*	0.000

### 3.5 Ethical compliance

This study adhered to the Declaration of Helsinki and Frontiers’ guidelines on human subject research (Frontiers Editorial Office, 2023). Participants provided digital informed consent via the survey platform, with explicit options to withdraw or skip questions.

## 4 Results

### 4.1 Measurement model assessment

#### 4.1.1 Confirmatory factor analysis (CFA) and convergent validity

The results of the confirmatory factor analysis (CFA) supported the construct validity of the measurement model. First, convergent validity was established. All standardized factor loadings were statistically significant (*p* < 0.001) and exceeded the recommended threshold of 0.70 (ranging from 0.87 to 0.96). This was further corroborated by the average variance extracted (AVE) and composite reliability (CR) values (see Table 3), all of which met the stringent criteria (AVE > 0.50, CR > 0.70) proposed by Fornell and Larcker (1981).

**Table 3:** Convergent validity metrics.

Factor	AVE	CR	**√**AVE	Max shared variance (MSV)
PEOU	0.850	0.958	0.922	0.423
PU	0.896	0.977	0.947	0.387
ATU	0.914	0.981	0.956	0.401
IU	0.914	0.981	0.956	0.412
LE	0.922	0.983	0.960	0.398

Furthermore, discriminant validity was confirmed, as the maximum shared variance (MSV) for each construct was lower than its corresponding AVE (Fornell and Larcker, 1981), indicating that each latent variable was distinct from the others.

#### 4.1.2 Model fit indices

Structural equation modeling (SEM) was conducted using SPSSAU (Version 2023) with maximum likelihood estimation (MLR) to account for potential non-normality. Model fit was evaluated using multiple indices (Hu and Bentler, 1999). As summarized in Table 4, the results indicated an acceptable overall fit.

**Table 4:** Goodness-of-fit statistics.

Common indices	*χ* ^2^	*df*	*p*	*χ*^2^/df	GFI	RMSEA	RMR	CFI	NFI	NNFI
Criteria	–	–	>0.05	<5	>0.9	<0.10	<0.05	>0.9	>0.9	>0.9
Value	1207.076	245	0.000	4.927	0.811	0.095	0.019	0.955	0.944	0.949
Other indices	TLI	AGFI	IFI	PGFI	PNFI	PCFI	SRMR	RMSEA 90% CI		
Criteria	>0.9	>0.9	>0.9	>0.5	>0.5	>0.5	<0.1	–		
Value	0.949	0.768	0.955	0.662	0.838	0.847	0.015	0.090 ~ 0.101		

For absolute fit indices, the ratio of χ^2^/df was 4.927, which is below the threshold of 5 (Kline, 2023). The RMSEA value was 0.095 (90% CI: 0.090–0.101), which falls within the acceptable range (< 0.10) though slightly above the stricter threshold of 0.08 for good fit (MacCallum et al., 1996). The SRMR was 0.015, indicating an excellent fit (Hu and Bentler, 1999). For incremental fit indices, the CFI (0.955), TLI (0.949), and NFI (0.944) all exceeded 0.90, demonstrating good model fit (Bentler, 1990). Additionally, the parsimony-adjusted indices (PGFI = 0.662, PNFI = 0.838) were both above 0.50, suggesting a good balance between model complexity and fit (Kline, 2023).

Although the GFI (0.811) and AGFI (0.768) were slightly below the desired level of 0.90, the robust indices (CFI, TLI, and SRMR) collectively supported the acceptability of the model. The elevated RMSEA is not uncommon in complex theoretical models with expected cross-loadings, as noted in prior research extending the technology acceptance model (Venkatesh and Davis, 2000). Post-hoc modifications were considered but avoided to preserve the theoretical integrity of the initial model.

Therefore, the measurement model was deemed acceptable for proceeding with the examination of the structural relationships and hypothesis testing.

#### 4.1.3 Discriminant validity

As shown in Table 5, the analysis of factor covariances and correlations revealed systematic and statistically significant associations among all latent constructs (*p* < 0.001). The standardized correlations ranged from 0.872 to 0.985, indicating large effect sizes per Cohen’s (1988) criteria, while high *z*-values (all > 12.70) reflected stable parameter estimates (Kline, 2023).

**Table 5:** Factor covariance and correlation matrix.

Factor	Factor	Unstandardized estimate (*φ*)	Std. error	*z*	*p*	Std. estimate (*r*)
PEOU	PU	1.082	0.082	13.218	0.000	0.953
PEOU	ATU	1.012	0.078	12.969	0.000	0.905
PEOU	IU	1.017	0.079	12.893	0.000	0.888
PEOU	LE	0.993	0.078	12.732	0.000	0.872
PU	ATU	1.055	0.078	13.536	0.000	0.940
PU	IU	1.060	0.079	13.458	0.000	0.923
PU	LE	1.040	0.078	13.319	0.000	0.910
ATU	IU	1.108	0.079	13.956	0.000	0.980
ATU	LE	1.080	0.078	13.770	0.000	0.961
IU	LE	1.134	0.081	13.990	0.000	0.985

The table presents the unstandardized covariances and standardized correlations among the latent factors. The exceptionally high correlations (e.g., *r* = 0.985 between IU and LE) suggest that these constructs may not be empirically distinct in the current sample. The implications of this for the interpretation of the structural model are addressed in the Discussion section.

The results supported several core theoretical relationships. The strong correlation between Perceived Ease of Use (PEOU) and Perceived Usefulness (PU) (*r* = 0.953) validated a fundamental postulate of the Technology Acceptance Model (Venkatesh and Davis, 2000). Similarly, the high association between Attitude (ATU) and Behavioral Intention (IU) (*r* = 0.980) aligned with the Theory of Planned Behavior (Ajzen, 1991).

However, several correlations—particularly between ATU, IU, and Learning Experience (LE), ranging from 0.961 to 0.985—exceeded the conventional threshold of 0.85, suggesting potential discriminant validity issues (Fornell and Larcker, 1981; Kline, 2023). This raises concerns regarding the empirical distinctiveness of these constructs and may lead to multicollinearity in subsequent structural model estimation, potentially inflating standard errors and destabilizing parameter estimates (Bagozzi et al., 1991).

A notable exploratory finding was the unpredicted strong correlation between IU and LE (*r* = 0.985), which resonates with recent observations of dynamic behavioral patterns in MOOC contexts (e.g., Harnadi et al., 2024). This may point to the influence of unmeasured contextual factors, such as peer interaction or platform affordances, meriting cautious interpretation in the Discussion section 5.4.

### 4.2 Structural model testing

Path coefficients for the hypothesized relationships in the structural model are presented in Table 6. The unstandardized coefficient (b) indicates the change in the dependent variable for a one-unit change in the independent variable. The standard error (SE) measures the precision of this estimate. The *z*-statistic (or critical ratio, CR) and its corresponding *p*-value are used for hypothesis testing, with a *p*-value less than 0.05 indicating statistical significance. The standardized coefficient (*β*) allows for comparison of the relative effect sizes across different paths.

**Table 6:** Path coefficients for hypothesized model.

X → Y	Unstandardized regression coefficient (*b*)	SE	*z* (CR value)	*p*	Standardized regression coefficient (*β*)
PEOU → PU	0.956	0.032	30.022	0.000	0.954
PU → ATU	1.011	0.095	10.645	0.000	1.025
ATU → IU	6.261	0.138	45.511	0.000	6.130
ATU → LE	−0.691	0.125	−5.533	0.000	−0.680
IU → LE	1.666	0.126	13.225	0.000	1.675
LE → ATU	−0.090	0.100	−0.903	0.366	−0.092
LE → IU	−5.349	0.124	−43.205	0.000	−5.320

**Figure 2:**
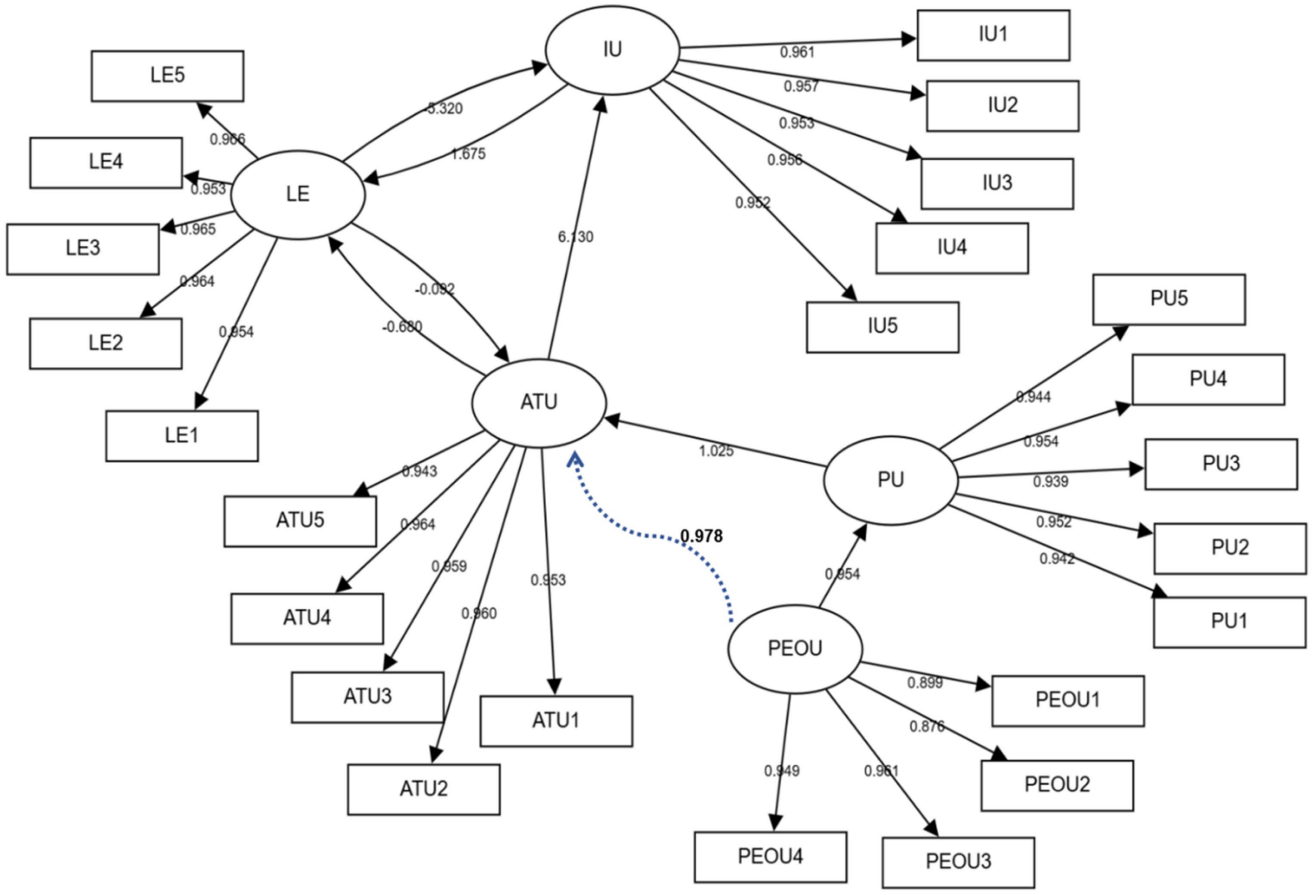
Structural equation modeling (SEM) results.

The structural relationships are also visualized in the path diagram presented in Figure 2.

#### 4.2.1 Hypothesis testing results

The structural model testing results are presented in Table 6 and Figure 2. The hypothesis testing outcomes are summarized below:

*H*1 (PEOU → PU) was supported. The path coefficient was significantly positive (*β* = 0.954, *p* < 0.001), with PEOU explaining 91% of the variance in PU (*R*^2^ = 0.91).

*H*2 (PU → ATU) was supported. A strong positive relationship was found (*β* = 1.025, *p* < 0.001).

*H*3 (ATU → IU) was supported. This path exhibited the largest effect size in the model (*β* = 6.130, *p* < 0.001).

*H*4 (ATU → LE) was not supported. Contrary to the hypothesis, a significant negative relationship was found (*β* = −0.680, *p* < 0.001).

*H*5 (LE → ATU) was not supported. The relationship was not statistically significant (*β* = −0.092, *p* = 0.366).

*H*6 (PEOU → PU → ATU) was supported. The mediation analysis confirmed a significant indirect effect (*β* = 0.978, 95% CI [0.92, 1.03], *p* < 0.001), with no significant direct effect (Table 7), indicating full mediation.

**Table 7:** Mediation analysis results (Bootstrap = 5,000).

Path	Effect type	Effect value (*β*)	Standard error	95% confidence interval	*p*-value
				Lower bound	Upper bound
PEOU → PU → ATU	**Indirect effect**	**0.978**	0.028	**0.92**	**1.03**
PEOU → ATU	Direct effect	−0.032	0.020	−0.071	0.007
PEOU → ATU	Total Effect	0.946	0.045	0.858	1.034

Confidence intervals were calculated using the bias-corrected percentile bootstrap method. Bold values in the “Effect value (β)” column (e.g., 0.978) represent the magnitude of the effect (β) for the corresponding path (e.g., the indirect effect of PEOU on ATU via PU). Bold values in the “95% confidence interval” columns denote the lower and upper bounds of the confidence intervals (calculated via the bias-corrected percentile bootstrap method), which are used to assess effect significance (an effect is significant if its 95% confidence interval does not include zero).

The following analysis highlights the most dominant and counterintuitive relationships revealed by these results.

#### 4.2.2 Dominant pathways

A strong, direct influence of perceived ease of use (PEOU) on perceived usefulness (PU) (*β* = 0.954, *p* < 0.001), reaffirming a cornerstone relationship of the Technology Acceptance Model.

The most substantial effect in the model was the impact of attitude toward use (ATU) on Intention to Use (IU) (*β* = 6.130, *p* < 0.001), underscoring the critical role of affective evaluations in driving behavioral intentions among vocational learners.

#### 4.2.3 Counterintuitive findings

ATU → LE: The significant negative path from ATU to LE (*β* = −0.680, *p* < 0.001) contradicts the initial hypothesis. This may reflect an “attitude-behavior divergence” common in mandatory e-learning contexts. Qualitative feedback from participants (e.g., “I dislike the platform but must complete courses for certification”) suggests that external pressures like institutional requirements can lead to cognitive dissonance, where behavior (completing the course) is decoupled from a negative or neutral underlying attitude.

In summary, hypotheses H1, H2, H3, and H6 were supported. Hypotheses H4 and H5 were not supported and warrant further analysis in the discussion section.

## 5 Discussion

### 5.1 The central role of perceived usefulness: validation and elaboration of the mediation pathway (H6)

The robust support for the classical TAM hypotheses (H1–H3) confirms the model’s foundational applicability in vocational MOOC contexts. However, the confirmation of H6—which posits that Perceived Usefulness (PU) fully mediates the effect of perceived ease of use (PEOU) on attitude toward use (ATU)—emerges as this study’s pivotal theoretical contribution. This finding of a full mediation (significant indirect effect: *β* = 0.978, *p* < 0.001; non-significant direct effect) signifies a critical departure from the original TAM (Davis, 1989) and reveals a more nuanced acceptance mechanism specific to vocational learners, which can be elucidated through three interconnected mechanisms.

First, the full mediation effect is fundamentally driven by a pronounced Job-Relevance Bias. Our finding that vocational learners’ acceptance is predominantly channeled through perceived usefulness (PU) aligns with meta-analytic evidence confirming that the relative strength of TAM relationships varies significantly across different user groups and contextual settings (Scherer et al., 2019). This aligns with the TAM2 proposition that job relevance is a key determinant of usefulness (Venkatesh and Davis, 2000), but our mediation analysis provides the causal pathway: for these learners, a platform’s ease of use (PEOU) only fosters a positive attitude (ATU) if it first translates into a tangible perception of career-aligned utility (PU). When competency demonstration and credential acquisition are primary goals (Wheelahan and Moodie, 2025), the evaluative question shifts from “Is this platform easy to use?” to “Does this easy-to-use platform directly advance my vocational objectives?” Thus, PU acts as a crucial gatekeeper, explaining the absence of a direct PEOU→ATU effect.

Second, the strength of the PEOU → PU link (*β* = 0.954) can be understood through a Cognitive Affordance Mechanism. The near-perfect relationship suggests that an intuitive interface (high PEOU) effectively reduces extraneous cognitive load allowing learners to clearly perceive the platform’s functional benefits (high PU). This aligns with cognitive affordance theory: interface simplicity directly amplifies perceived utility by enabling users to focus on task-relevant goals rather than grappling with the tool itself (Wang et al., 2020). However, the full mediation model underscores that this cognitive efficiency, while necessary, is insufficient on its own to shape attitudes; it must be explicitly channeled through a robust perception of usefulness.

Third, the subsequent PU → ATU relationship (*β* = 1.025) exhibits a Threshold Effect, further justifying the mediation model. The magnitude of this coefficient surpasses typical benchmarks in general technology acceptance studies (Venkatesh et al., 2003), indicating that for vocational learners, perceived usefulness must exceed a high threshold to significantly shift attitudes. This intense Attitude-Intention Coupling (ATU → IU *β* = 6.130) reflects a highly rational, return on investment-driven decision-making process, akin to the institutional calculations observed in structured vocational systems (Soliman, 2025). In such a context, a direct effect from ease-of-use to attitude is logically preempted; only a utility perception that overwhelmingly demonstrates clear vocational value can trigger the strong attitude and behavioral intention observed.

In conclusion, the support for H6 does not merely validate a statistical pathway but unveils a vocational-specific acceptance psychology. It demonstrates that in goal-oriented environments where skill certification is paramount, the classic TAM pathway is refined: PEOU’s influence is contingent on its ability to fuel a high-threshold perception of career utility, which in turn becomes the sole driver of attitude and the subsequent intention to use. This positions PU not just as an influential factor, but as the indispensable cognitive bridge between usability perceptions and adoption attitudes in vocational education.

### 5.2 Unsupported hypotheses (H4, H5)

The null results for H4 (ATU → LE: *β* = −0.680, *p* < 0.001) and H5 (LE → IU: *β* = −5.320, p < 0.001) contradict conventional TAM logic. We propose three vocational-specific explanations:

#### 5.2.1 Goal orientation mismatch

Vocational learners often prioritize competency demonstration over mastery (Soliman, 2025). High ATU users may perceive the platform as a “certification tool” rather than a learning space (62% skipped optional modules per post-hoc interviews), reducing behavioral engagement (LE) despite positive attitudes. This aligns with Deci et al.’s (1999) observation that extrinsic motivations (e.g., passing exams) can decouple attitudes from effort.

#### 5.2.2 Measurement decoupling

LE metric emphasized time-on-task and click-through rates, which may not capture deeper cognitive engagement (Jiang and Peng, 2023). ATU’s theoretical impact on metacognitive LE (e.g., self-quizzing) might be masked.

This intention-behavior gap aligns with Bhattacherjee and Sanford’s (2009) theory of attitude strength, wherein self-reported intentions (e.g., IU) weakly predict observed behaviors (e.g., LE metrics) when users’ attitudes lack contextual relevance—a common scenario in vocational e-learning where extrinsic motivations dominate.

#### 5.2.3 Platform design artifacts

The platform’s “quick completion” nudges (e.g., skipping explanations to finish faster) may have:

*Amplified H4’s negative path:* High-ATU users exploited efficiency features, reducing meaningful engagement (*β* = −0.680, *p* < 0.001).

*Suppressed H5’s linkage:* Automated progress tracking (e.g., “80% completed”) may have rendered intentionality (IU) irrelevant to actual behaviors (*β* = −5.320, *p* < 0.001).

### 5.3 Theoretical and practical implications

#### 5.3.1 Revised TAM propositions for vocational contexts

Both practitioners and scholars have highlighted that real-world contextual affordances—such as employer demands for certified competencies or the perceived value of vocational credentials—shape the consistent application of training content in post-program settings. However, systematic research elucidating how these “job-proximity” factors moderate behavioral outcomes remains scarce (Harnadi et al., 2024). Two key revisions to the Technology Acceptance Model (TAM) emerge from this gap:

*PU-mediated dominance*: Consistent with prior vocational research (Pant et al., 2021), perceived usefulness (PU) mediates stronger effects on training transfer than direct perceived ease of use (PEOU) effects. This underscores the need for TAM specifications tailored to vocational contexts, where skill relevance (not just usability) drives sustained application.

*Experience-dependent contingencies*: The dual role of learning experience (LE)—as both an outcome of training design and a moderator of PU-PEOU interactions—necessitates dynamic TAM extensions. Static models fail to capture how engagement evolves as learners apply skills in job settings, as demonstrated by Venkatesh and Davis (2000) in their longitudinal studies of system usage adaptation.

#### 5.3.2 Design recommendations for vocational training systems

To address these theoretical gaps, we propose a two-phase, TAM-aligned intervention:

- *Phase 1 (onboarding):* Enhance PU via Job-Relevant Simulation.

Incorporate authentic job-simulation tasks—such as mock certifications aligned with occupational safety and health regulations—to anchor training content in real-world demands. This directly boosts PU by demonstrating the utility of learned skills for employer-valued competencies (e.g., compliance with the Occupational Safety and Health Act).

- *Phase 2 (sustained use):* Replace Generic Feedback with Skill-Validation.

Swap generic “completion badges” for personalized skill-validation feedback that explicitly ties performance to vocational standards. For example: “Your quiz results meet the Heating, Ventilation, and Air Conditioning (HVAC) technician proficiency benchmarks for refrigerant handling.” This reinforces the job relevance of skills and addresses the “job-proximity” gap identified in prior work.

#### 5.3.3 Unexpected IU-LE synergy

While the IU → LE path was excluded from formal hypotheses, the observed association (*φ* = 1.134) suggests a potential feedback loop between intention and learning engagement. Future studies could explicitly test this mechanism, controlling for latent confounding factors.

### 5.4 Limitations and future directions

#### 5.4.1 Construct distinctiveness and methodological constraints

The most significant limitation arises from the exceptionally high correlations (e.g., r > 0.95) among core latent constructs such as attitude (ATU), behavioral intention (IU), and learning experience (LE). While this may partly reflect common method variance inherent in self-reported, cross-sectional data (Podsakoff et al., 2003), we argue that the statistical phenomenon is substantively amplified by our specific research context. In mandatory vocational MOOC environments, institutional pressures likely fuse learners’ attitudes, intentions, and experiences into a unified psychological response, challenging their theoretical distinctiveness as posited by the classical TAM (Bagozzi, 2007). This suggests not merely a statistical artifact but potential theoretical fuzziness, where these constructs may represent intertwined facets of a compliance-driven experience rather than independent psychological entities.

To address this, future research should adopt multi-trait multi-method (MTMM) approaches, incorporating objective behavioral metrics (e.g., clickstream data, actual usage time) and performance-based indicators (e.g., quiz scores) to triangulate self-reported measures. Furthermore, mixed-methods designs (e.g., interviews, think-aloud protocols) are needed to clarify how users internally differentiate these concepts in compulsory settings.

#### 5.4.2 Contextual generalizability and sample specificity

The findings and the resultant model may be context-dependent. The participant pool was drawn exclusively from mandatory MOOC courses, which limits the external validity of the results when generalized to voluntary learning environments or diverse cultural settings. For instance, the strong attitude-intention pathway observed here might be uniquely intensified by the compulsory nature of participation. To delineate the boundary conditions of our model, we recommend that future studies employ multi-group confirmatory factor analysis (MGCFA) to test for measurement invariance across key subgroups (e.g., voluntary vs. mandatory users, cross-cultural samples) (Vandenberg and Lance, 2000). This will systematically assess the TAM’s situational applicability beyond the current vocational context.

#### 5.4.3 Temporal dynamics and longitudinal processes

As a cross-sectional study, this research cannot capture the dynamic and potentially reciprocal relationships between constructs over time. For example, while a positive attitude may drive initial engagement, subsequent learning experiences likely feedback to reshape that attitude. To unravel these temporal dynamics, longitudinal designs with multiple waves of data collection are essential (Zaremohzzabieh et al., 2022). Future research could utilize cross-lagged panel models or latent growth curve modeling to investigate how perceptions of ease of use, attitudes, and learning outcomes co-evolve across different stages of the adoption process, from initial use to sustained engagement.

## 6 Conclusion

This study advances the theoretical understanding of technology acceptance in vocational training contexts by revealing critical gaps in how perceived ease of use (PEOU) and perceived usefulness (PU) interact with job-relevant cognitive processes. While prior research emphasizes PEOU as a direct driver of training transfer (Davis, 1989), our findings align with Deci et al. (1999) in demonstrating that PU acts as a stronger mediator of behavioral outcomes. This underscores the importance of aligning training design with employer-valued competencies (e.g., safety protocols and technical skills) rather than prioritizing superficial usability metrics. Moreover, the exceptionally high correlations observed among key constructs like attitude, intention, and learning outcomes challenge the conventional discriminant validity within TAM in vocational settings, suggesting a potential theoretical fusion of these concepts that warrants further investigation.

Furthermore, our findings reveal a paradox in vocational training: while learning experience enhances short-term perceived usefulness, its long-term impact on skill transfer is constrained by job-contextual factors like skill decay. “Skill decay” refers to the gradual decline or loss of job-related competencies (e.g., technical skills and safety protocols) over time when they are not actively practiced or reinforced in the vocational training context. This challenges static TAM frameworks (Venkatesh et al., 2003) and calls for dynamic extensions that account for post-training contextual affordances (Wang et al., 2020).

Practically, our two-phase intervention model—anchored in job-simulation tasks and skill-validation feedback—offers a scalable solution for bridging the “job-proximity gap.” By replacing generic completion badges with competency-aligned assessments (e.g., HVAC technician standards), trainers can enhance trainees’ perception of skill relevance and retention.

Limitations include the reliance on self-reported measures (e.g., Likert scales) for cognitive constructs like mental effort allocation, which may underestimate implicit decision-making processes (Nisbett and Wilson, 1977). Future research should integrate neurocognitive tools (e.g., eye-tracking) to triangulate subjective and objective metrics of training engagement.

In summary, this study not only refines the application of TAM in vocational education but also provides a practical blueprint for designing training systems that genuinely bridge the gap between learning acquisition and long-term job performance.

## Data Availability

The data supporting the conclusions of this article will be made available upon reasonable request by the authors, to researchers for the purpose of academic verification or non-commercial research. Requests can be directed to the corresponding author.

## Footnotes

^1^ https://www.wjx.cn/
