# Correction: Exploring learner acceptance and experience in MOOCs among higher vocational students from a TAM perspective: a case study of college English course

**DOI:** 10.3389/fpsyg.2026.1788759

**Published:** 2026-02-02

**Authors:** Shuhua Hou

**Affiliations:** School of Higher Vocational Education, The Open University of Sichuan, Chengdu, China

**Keywords:** technology acceptance model (TAM), MOOCs, learning experience, higher vocational education, structural equation modeling (SEM)

A reference was erroneously written in the published article. It was captured as:

Guedes, K. K. D., Davis, H. C., and Schulz, J. (2022). Integrating MOOCs into traditional higher education modules: a MOOC-based blend framework. *Res. Learn. Technol*. 30:2702. doi: 10.25304/rlt.v30.2702

The correct reference appears below:

de Lima Guedes, K. K., Davis, H. C., and Schulz, J. (2022). Integrating MOOCs into traditional higher education modules: a MOOC-based blend framework. *Res. Learn. Technol*. 30:2702. doi: 10.25304/rlt.v30.2702

The previous in-text citation of “Guedes et al., 2022” was updated to “de Lima Guedes et al., 2022”.

The original version of this article has been updated.

