# Supplementary material for: Exploring learner acceptance and experience in MOOCs among higher vocational students from a TAM perspective: a case study of college English course
Source: Front Psychol. 2025 Nov 28;16:1643166. doi: 10.3389/fpsyg.2025.1643166 (PMC12698547; doi:10.3389/fpsyg.2025.1643166)
Supplement: Supplementary file 1 [file Table_1.docx]

| Standardized CFA Loadings | | | | | | | |
| --- | --- | --- | --- | --- | --- | --- | --- |
| Factor | variables | Unstandardized Factor Loadings | Std. Error | *z* (CR) | *p* | Std. Estimate | SMC |
| PEOU | PEOU1 | 1.000 | - | - | - | 0.899 | 0.808 |
| PEOU | PEOU2 | 0.961 | 0.035 | 27.838 | 0.000 | 0.876 | 0.767 |
| PEOU | PEOU3 | 1.030 | 0.029 | 36.068 | 0.000 | 0.962 | 0.925 |
| PEOU | PEOU4 | 1.006 | 0.029 | 34.607 | 0.000 | 0.949 | 0.900 |
| PU | PU1 | 1.000 | - | - | - | 0.942 | 0.887 |
| PU | PU2 | 0.995 | 0.023 | 42.916 | 0.000 | 0.952 | 0.907 |
| PU | PU3 | 0.970 | 0.024 | 40.303 | 0.000 | 0.939 | 0.882 |
| PU | PU4 | 1.000 | 0.023 | 43.262 | 0.000 | 0.954 | 0.910 |
| PU | PU5 | 0.984 | 0.024 | 41.260 | 0.000 | 0.944 | 0.892 |
| ATU | ATU1 | 1.000 | - | - | - | 0.953 | 0.909 |
| ATU | ATU2 | 1.015 | 0.021 | 47.989 | 0.000 | 0.960 | 0.922 |
| ATU | ATU3 | 1.001 | 0.021 | 47.692 | 0.000 | 0.959 | 0.920 |
| ATU | ATU4 | 0.984 | 0.020 | 49.217 | 0.000 | 0.964 | 0.929 |
| ATU | ATU5 | 0.987 | 0.023 | 43.523 | 0.000 | 0.943 | 0.889 |
| IU | IU1 | 1.000 | - | - | - | 0.961 | 0.924 |
| IU | IU2 | 0.977 | 0.020 | 49.391 | 0.000 | 0.957 | 0.915 |
| IU | IU3 | 0.996 | 0.021 | 48.378 | 0.000 | 0.953 | 0.909 |
| IU | IU4 | 0.970 | 0.020 | 49.246 | 0.000 | 0.956 | 0.914 |
| IU | IU5 | 0.988 | 0.021 | 48.058 | 0.000 | 0.952 | 0.907 |
| LE | LE1 | 1.000 | - | - | - | 0.954 | 0.909 |
| LE | LE2 | 0.997 | 0.020 | 49.470 | 0.000 | 0.965 | 0.930 |
| LE | LE3 | 1.018 | 0.021 | 49.586 | 0.000 | 0.965 | 0.931 |
| LE | LE4 | 0.982 | 0.021 | 46.160 | 0.000 | 0.953 | 0.908 |
| LE | LE5 | 1.001 | 0.020 | 49.749 | 0.000 | 0.965 | 0.932 |
| Note: PEOU= Perceived Ease of Use, PU= Perceived Usefulness, ATU= Attitude Toward Use, IU= Intention to Use, LE= Learning Experience. | | | | | | | |
